# Impact of advanced endoscopy training on colonoscopy quality and efficiency

**DOI:** 10.1002/deo2.70027

**Published:** 2024-10-12

**Authors:** Takashi Watanabe, Tatsuro Murano, Hiroaki Ikematsu, Kensuke Shinmura, Masashi Wakabayashi, Nobuhisa Minakata, Sasabe Maasa, Tomohiro Mitsui, Hiroki Yamashita, Atsushi Inaba, Hironori Sunakawa, Keiichiro Nakajo, Tomohiro Kadota, Tomonori Yano

**Affiliations:** ^1^ Department of Gastroenterology and Endoscopy National Cancer Center Hospital East Chiba Japan; ^2^ Course of Advanced Clinical Research of Cancer Juntendo University Graduate School of Medicine Tokyo Japan; ^3^ Biostatistics Division Center for Research Administration and Support National Cancer Center Chiba Japan

**Keywords:** adenoma, colonoscopy, fellowships, retrospective studies, scholarships

## Abstract

**Objectives:**

Few reports have detailed improvements in the quality of colonoscopies with continuous training post‐fellowship completion. We examined the changes in colonoscopy performance among trainees during our advanced endoscopy training program.

**Methods:**

Screening or surveillance colonoscopies performed by 11 trainees who participated in our 3‐year advanced endoscopy training program between April 2015 and March 2020 were retrospectively analyzed. Quality and efficiency metrics of colonoscopies were evaluated annually.

**Results:**

Altogether, 297, 385, and 438 colonoscopies were enrolled in the first, second, and third training years, respectively. The mean insertion times were 8.6, 7.6, and 6.9 min in the first, second, and third training years, respectively, with significant improvement from the first to second year (*p* = 0.03) and from the first to third year (*p* < 0.01). The adenoma detection rate, proximal adenoma detection rate, and mean number of adenomas per patient exhibited a tendency to improve annually; however, the difference was not significant. Polypectomy efficiency was 10.5%, 11.2%, and 13.0%, with significant improvements from the first to third year (*p* < 0.01) and from the second to third year (*p* = 0.02). Insertion time and polypectomy efficiency showed significant improvements, especially among trainees experienced with <500 colonoscopies.

**Conclusions:**

Through our advanced endoscopy training program, there has been an improvement in the quality and efficiency of colonoscopy for trainees who have completed their fellowships, particularly those with <500 colonoscopies.

## INTRODUCTION

Colonoscopy is widely accepted as the gold standard for colorectal lesion screening and has demonstrated a reduction in the morbidity and mortality associated with colorectal cancer.^1^, ^2^ A major factor affecting the quality of colonoscopies is endoscopists’ proficiency.^3^ Consequently, developing training programs that certify trainees as competent in performing colonoscopies is important. In Western and Asian countries, colonoscopy training, integrated into fellowship programs for general gastroenterologists or surgeons, has improved colonoscopy quality.^4^, ^5^, ^6^ However, concerns remain regarding whether colonoscopy quality among fellowship graduates has reached the level of attending physicians owing to the restricted endoscopy training times because of other service demands.^7^

Over the past decade, some countries have implemented extended training programs to facilitate the transition from fellow graduates to independent endoscopists.^8^, ^9^ To date, although there have been some reports on changes in therapeutic colonoscopy, such as endoscopic mucosal resection and endoscopic submucosal dissection (ESD), during advanced endoscopy training programs,^10^, ^11^ there is limited information regarding the impact of these advanced programs on changes in colonoscopy quality. Therefore, we aimed to examine the changes in colonoscopy performance among trainees during our advanced endoscopy training program.

## METHODS

### Study population and data collection

We conducted a retrospective analysis of colonoscopies performed by all 11 trainees who participated in an advanced endoscopy training program at the National Cancer Center Hospital East between April 2015 and March 2020; four attending endoscopists supervised the training. To assess the changes in colonoscopy quality and efficiency at different training levels, we included screening or surveillance colonoscopies performed by trainees in their first six months of first year, and second 6 months of second and third year, as well as those performed by attending endoscopists. The exclusion criteria of cases for assessment were as follows: (1) prior colorectal surgery, (2) a history of chemoradiotherapy or chemotherapy for colorectal cancer, (3) colonoscopy for preoperative evaluation of advanced cancer, (4) polyposis cases such as familial adenomatous polyposis and Lynch syndrome, and (5) colonoscopies with overall poor preparation rated more than “poor” on the Aronchick bowel preparation scale. Details regarding the colonoscopies excluded by these criteria are shown in Table S1. Data on insertion and withdrawal times, success of cecal intubation, number of resected colorectal lesions, and their clinicopathological characteristics were extracted from electronic medical records.

### Advanced endoscopy training program

Since 2013, our department has been offering a 3‐year training program specializing in advanced diagnostic and therapeutic endoscopy in the gastrointestinal field (Table 1). In Japan, trainees usually complete a general gastroenterology fellowship program during their first 2–4 years as gastroenterologists. During this time, they encounter simple colonoscopy cases and perform basic endoscopic procedures such as biopsy and polypectomy. Our program was developed for endoscopists who have completed their general gastroenterology fellowship. Although we have not created a strict definition of fellowship completion, we recommend that trainees should apply to our program after completing at least 2–3 years of a general gastroenterology fellowship. For colonoscopy procedures, the final goal is to acquire expertise in effective insertion technique, independently conduct polypectomy or endoscopic mucosal resection based on the appropriate diagnosis using magnifying endoscopy, and perform advanced therapeutic procedures such as ESD, dilatation, and stent insertion. In our program, first‐ and second‐year trainees perform colonoscopies under the supervision of attending endoscopists and receive verbal instructions and technical support as needed. Additionally, by performing colonoscopies alongside attending endoscopists in the same endoscopic room, trainees can closely observe the procedures performed and practice them immediately. Third‐year trainees independently perform colonoscopies and can seek support from attending endoscopists when necessary. In the first year of training, prior to performing a colonoscopy, trainees learn about the insertion method using the axis‐keeping shortening technique through lectures led by attending endoscopists and hands‐on practice with the Colonoscope Training Model (Kyoto Kagaku Co., Ltd.). Moreover, through lectures and regular weekly conferences held throughout the year, trainees learn how to detect lesions, perform magnifying observations using image‐enhancing endoscopes, and make qualitative and quantitative diagnoses based on endoscopic findings in actual cases. The educational features of our program are summarized in Table 1.

**TABLE 1:** Features of our training program.

**Policy**	All years	Insertion method using the axis‐keeping shortening technique for all cases Diagnosis with magnifying endoscopy for all neoplastic lesions Resect all suspected adenomatous lesions during colonoscopies (clean colon)
**Orientation**	Prior to first year (2 weeks)	Lecture about basic knowledge (insertion and detection technique, diagnosis methods, and treatment choices) by attending endoscopists Observation of colonoscopy by attending endoscopists Intensive insertion practice with Colonoscope Training Model
**One‐on‐one instruction**	First and second years	Perform colonoscopy in the same endoscopic room with the attending endoscopist Observe the attending procedures up close and subsequently perform colonoscopies under the supervision of an attending endoscopist
**Weekly conference**	All years	Reading endoscopic findings of ESD‐eligible lesions Close examination of endoscopically treated lesions for correspondence between magnifying endoscopic findings and histopathology
**Advanced procedure**	Third year	Perform advanced endoscopic procedures such as transanal decompression tube, colorectal stent and ESD

### Colonoscopy procedure

We used either the EVIS LUCERA ELITE CLV‐290 (Olympus Corporation) or LASERIO (Fujifilm Corporation) as the light source and video processor, and predominantly relied on a magnifying colonoscope (PCF‐H290ZL/I, CF‐HQ290, CF‐H290ECI; Olympus Corporation and EC‐L590ZP, EC‐L600ZW7; Fujifilm Corporation). All patients underwent standard bowel preparation using either a polyethylene glycol electrolyte lavage solution or oral sodium phosphate. Before the examination, butylscopolamine or glucagon was injected intravenously as an antispasmodic medication. We used analgesics, such as meperidine and midazolam, only upon the patient's request or in patients who experienced severe pain in their previous endoscopies.

For colonic intubation, the axis‐keeping shortening technique was initially attempted in all cases.^12^ We had an agreement that if a patient complained of severe pain during insertion or if it took longer than 10 min, especially during sigmoid colon insertion, the trainee was replaced by an attending endoscopist. Cecal intubation was performed for screening and surveillance in all cases. Even in cases where trainees were substituted by an attending during insertion, the trainees completed the endoscopy at the time of removal. When a polyp was detected, the endoscopists recorded its location, morphology, and size prior to resection. Location was defined as the proximal colon when polyps were present in the cecum, ascending colon, or transverse colon. For any detected lesion, we decided on a treatment plan after a qualitative diagnosis of the lesion using magnifying endoscopic or chromoendoscopic observation. We followed a general policy for resecting all suspected adenomatous lesions during all colonoscopies. Resection and discard strategies were not implemented in any procedure.

### Outcome definitions

We evaluated the insertion time and cecal intubation rate to gauge the proficiency of the insertion technique, and adenoma detection rate (ADR), proximal ADR, and mean number of adenomas per patient (MAP) as indices of the ability to identify and diagnose neoplastic lesions. Polypectomy efficiency (PE) was assessed as an indicator of procedural efficiency during colonoscopy.^13^ We compared outcomes for each of these indicators annually and those of the attending endoscopists.

Insertion time was the time to reach the cecum from scope insertion. Cecal intubation rate was the percentage of colonoscopies reaching the cecum independently. ADR and proximal ADR were percentages of colonoscopies detecting at least one adenoma or cancer in the total and proximal colon. MAP was the average number of lesions detected per colonoscopy. PE was calculated as (polyps resected/withdrawal time) × 100, indicating procedure efficiency rather than diagnostic competence. Insertion times were primarily obtained from the endoscopy reports. When the insertion time was not documented in the report, we referred to the endoscopic images, which often included a timer that indicated when the insertion process was started. However, because of the retrospective nature of the study, the insertion time could not be determined for 215 (15%) cases. Cases with missing data regarding the insertion time were excluded from the insertion time analysis and PE analysis.

### Statistical analysis

We employed the student's *t*‐test for continuous variables and the chi‐squared test or Fisher's exact probability test for categorical variables. In the analysis of the changes in colonoscopy performance over time, we conducted all two‐group comparisons made between each training year. A two‐sided *p*‐value <0.05 was considered statistically significant. Statistical analyses were performed using GraphPad Prism 9.1.0.

## RESULTS

### Baseline trainee and colonoscopy characteristics

The characteristics of the 11 participants are shown in Table 2. These trainees were predominantly male (90.9%) and right‐handed. At the start of training, the trainees had a median of 4 years of endoscopic experience. Six trainees had previous experience with 500 or more colonoscopies, whereas five trainees had performed less than 500 but at least 150 colonoscopies. None of the trainees were beginners with fewer than 150 colonoscopies. Baseline patient and colonoscopy characteristics are shown in Table 3. The total number of colonoscopies analyzed was 297, 385, 438, and 273 for the first‐, second‐, and third‐year trainees, and attending physicians, respectively. On average, 27, 35, 39.8, and 68.3 colonoscopies per person were performed by the first‐, second‐, and third‐year trainees, and attending physicians, respectively. No significant differences were observed in age, sex, or withdrawal time between the groups, except for a slightly longer withdrawal time for the attending physician. The percentage of screening colonoscopies tended to increase with successive training years, while the number of screening and surveillance colonoscopies was nearly equal among the attending physicians.

**TABLE 2:** Trainee characteristics.

Total number of trainees	11
Male sex (%)	10 (90.9)
Right‐handed (%)	11 (100)
Years of endoscopy experience at the start of training (range)	4 (2–7)
Training hospital setting	
Country	10
University	1
The number of colonoscopy experiences (%)	
≧500	6 (54.5)
<500, ≧150	5 (45.5)
<150	0 (0)

**TABLE 3:** Patients and colonoscopy characteristics by each training year and attending.

	First‐year trainee (*n* = 11)	Second‐year trainee (*n* = 11)	Third‐year trainee (*n* = 11)	Attending (*n* = 4)
Total number of colonoscopies	297	385	438	273
The mean number of colonoscopies per trainee or attending	27	35	39.8	68.3
Age (range)	68 (30–88)	68 (24–86)	68 (26–89)	70 (24–86)
Male sex, number (%)	205 (61.2)	271 (65.9)	269 (61.1)	189 (69.2)
Indication, number (%)				
Screening	151 (50.8)	210 (54.5)	269 (61.4)	149 (54.6)
Surveillance	146 (49.2)	175 (45.5)	169 (38.6)	124 (45.4)
Mean withdrawal time, min (range)				
Polypectomy performed	20.3 (11–29)	20.3 (11–38)	20.8 (10–38)	23.9 (6–57)
No polypectomy	12.2 (7–47)	12.6 (7–20)	13.0 (5–24)	14.5 (6–36)

### Change in quality metrics of colonoscopy during training

**FIGURE 1:**
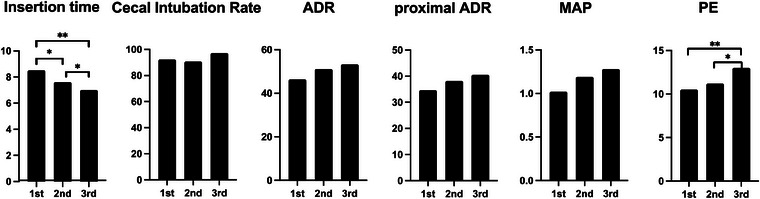
Change in colonoscopy metrics over the training period. The asterisk (*) indicates *p* < 0.05, and the asterisks (**) indicate *p* < 0.01 for comparison between years.

The changes in colonoscopy indices calculated for each trainee year are shown in Figure 1. The average insertion time significantly improved from the first (8.5 min) to the second (7.6 min) trainee year (*p* < 0.05), as well as from the first to third (7.0 min) trainee year (*p* < 0.01). The cecal intubation rate remained high at >90% throughout the training period. Colonoscopy indices, such as ADR was 46.4%. 51.1%, and 53.2%, proximal ADR was 34.6%, 38.2%, and 40.5%, and MAP was 1.02, 1.19, and 1.28, in the first, second, and third trainee years, respectively. Although no significant differences were observed between any two trainee years, there was a consistent yearly improvement in each metric. Significant improvement was demonstrated in PE from the first (10.5%) to the third (13.0%) year (*p* < 0.01) and from the second (11.2%) to the third year (*p* < 0.05).

### Comparison of colonoscopy indices between third‐year trainees and attending physicians

To determine whether our advanced endoscopy training enabled endoscopic competence on par with the attending physicians, the colonoscopy quality metrics were compared between the third‐year trainees and the attending physicians (Table 4).

**TABLE 4:** Comparison of colonoscopy indicators between third‐year trainee and attending physician.

	Third‐year trainee	Attending	*p*‐Value
Insertion time, average (range)	7.03 (1–29)	5.20 (1–35)	<0.01
ADR (%)	53.2	59.9	0.26
Proximal ADR (%)	40.5	44.6	0.65
MAP (%)	1.28	1.5	<0.01
PE	12.8	11.9	0.32

Abbreviations: ADR, adenoma detection rate; MAP, mean number of adenomas per patient; PE, polypectomy efficiency.

The results indicated that attending trainees outperformed third‐year trainees in terms of average insertion time (5 min, *p* < 0.01) and MAP (1.5, *p* < 0.01), whereas no significant differences were found in ADR, proximal ADR, and PE.

### Sub‐analysis of colonoscopy indices by the indication of colonoscopy

**FIGURE 2:**
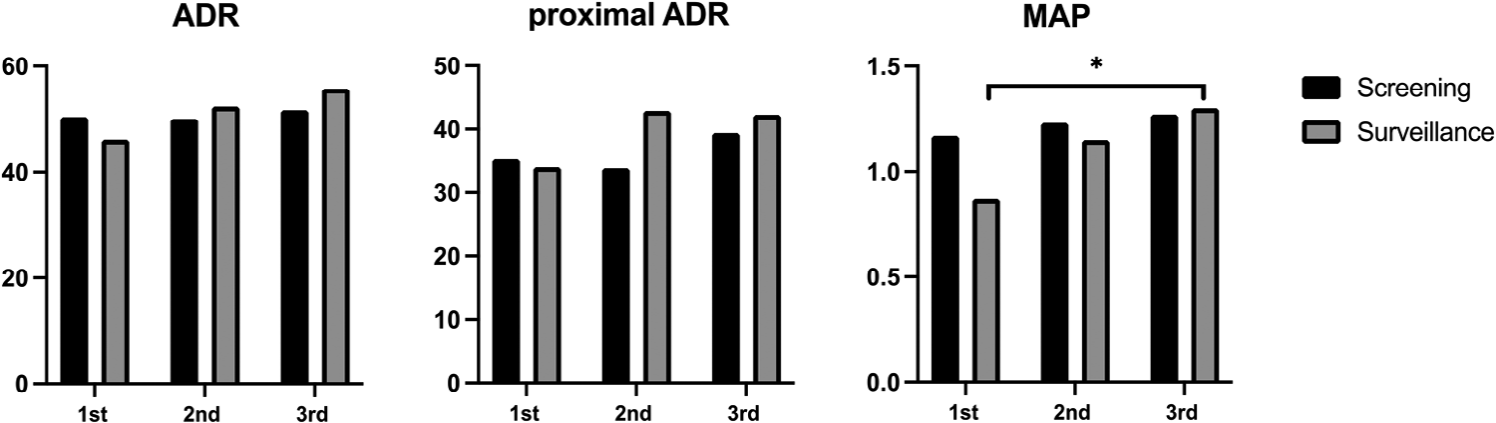
Polyp detection indicators by sub‐analysis divided into surveillance and screening. The asterisk (*) indicates *p* < 0.05 for comparison between each year.

As differences in ADR between surveillance and screening colonoscopies have been reported,^14^ we conducted a sub‐analysis of indices regarding neoplastic lesion detection and diagnosis. The evaluation of ADR, proximal ADR, and MAP stratified by screening and surveillance colonoscopy is shown in Figure 2. No significant changes were found in ADR and proximal ADR with training levels for either surveillance or screening colonoscopy. However, for MAP, although there were no significant changes in surveillance colonoscopy, a significant improvement was noted from the first (1.17) to the third trainee (1.27) year (*p* < 0.05) in screening colonoscopy.

### Change in colonoscopy indices based on colonoscopy experience at the commencement of training

**FIGURE 3:**
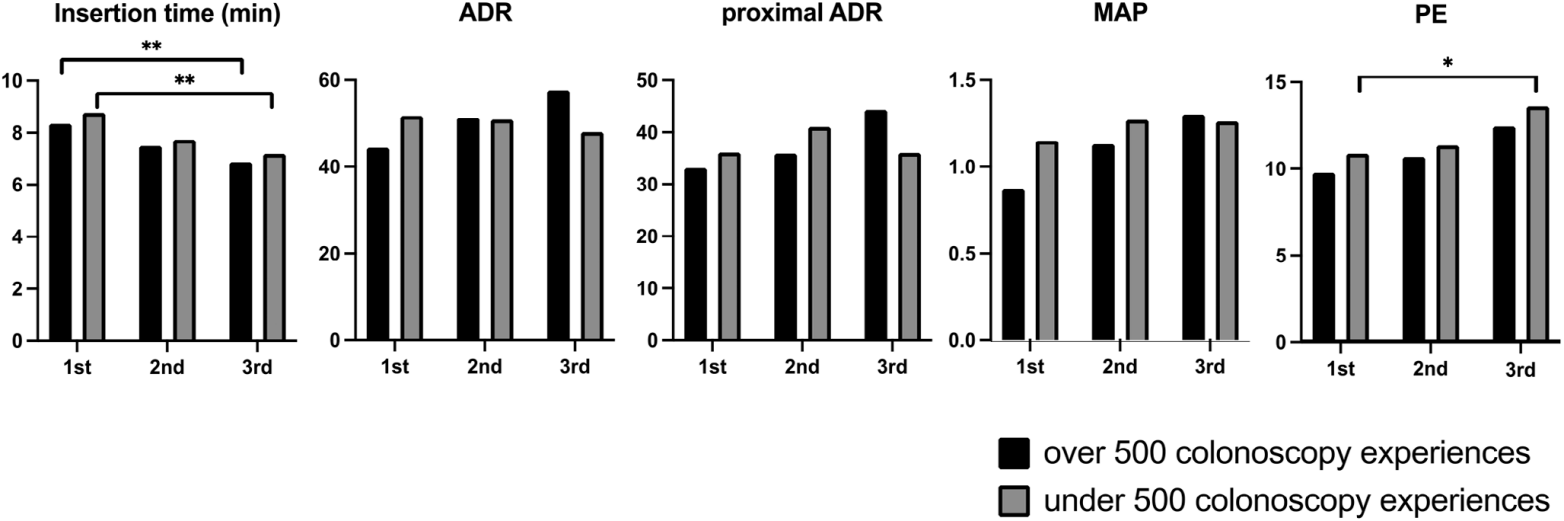
Change in colonoscopy indicators by prior colonoscopy experience at the outset of training. The asterisk (*) indicates *p* < 0.05, and the asterisks (**) indicate *p* < 0.01 for comparison between years.

Given the disparity in the number of colonoscopies trainees had performed at the outset of our training program (Table 2), we investigated whether there was any difference in the change in colonoscopy indices depending on the trainees’ previous colonoscopy experiences. The change in each colonoscopy index is shown in Figure 3 where the number of experienced colonoscopies is stratified into less than and more than 500. Notably, for insertion time, a significant improvement was observed from the first to the third trainee year, regardless of the number of experienced colonoscopies. Similar to the pre‐stratification analysis (Figure 1), there was little variation in the ADR, proximal ADR, or MAP according to the number of previously performed colonoscopies. A significant improvement from the first to the third year was found only for trainees with less than 500 colonoscopy experiences.

## DISCUSSION

This retrospective observational study analyzed the changes in the quality and efficiency of colonoscopies performed by trainees in our advanced endoscopic training program. We evaluated previously reported quality measures of colonoscopy, such as insertion time, ADR, MAP, and PE, a measure of procedural efficiency, over consecutive years. We found that our advanced endoscopic training program significantly improved these measures with each passing year, especially the insertion time, PE, and MAP in surveillance colonoscopy. The stratified analysis revealed significant improvements in insertion time and PE among trainees with prior experience of <500 colonoscopies during their fellowship. This study underscores the effectiveness of our advanced endoscopy training program in improving the quality and efficiency of colonoscopy over time for post‐fellowship trainees, particularly those with fewer than 500 colonoscopy experiences.

Guidelines in each country stipulate the requisite number of colonoscopies to achieve sufficient proficiency during a fellowship. For example, in the United States, 140 colonoscopies are recommended, while in the United Kingdom, 200 colonoscopies are recommended.^2^, ^3^, ^15^, ^16^, ^17^ However, these target numbers, calculated based on the cecal intubation rate as an indicator, do not necessarily guarantee trainee proficiency. Consequently, it has been proposed that competence standards should be established, considering all aspects of colonoscopy: insertion, detection, and treatment. In fact, data from South Korea indicate that a minimum of 500 cases is required to be able to independently perform colonoscopy insertion, treatment, and hemostasis in more than 90% of the cases, highlighting the need for continuous advanced training even after fellowship completion.

Several aspects of our advanced endoscopy fellowship program, offered since 2015, are worth mentioning. First, trainees enter the program with a strong foundation in essential endoscopic skills, especially the fundamental insertion technique. Second, during the first and second years of training, the trainees conduct examinations under the supervision of an attending physician. This environment empowers trainees to learn from these experienced practitioners on a one‐on‐one basis and promptly put their learning into practice. Although there have been numerous reports on the improvements in ESD and other treatment skills among trainees participating in advanced endoscopy fellowships.^10^, ^11^ To the best of our knowledge, this is the first study to elucidate the changes in colonoscopy competence. We believe that evaluating the progression of trainee skills over time in our advanced fellowship program will aid not only in designing similar endoscopy programs in the future but also in assessing the long‐term learning curve from novice to expert.

Our study showed a trend toward year‐to‐year improvements across all metrics of insertion, lesion detection, and procedural efficiency. Improvement in the insertion time was particularly significant, with an average insertion time of 7 min in the third year. This suggests the efficacy of the insertion method employed, known as the axis‐keeping shortening technique.^12^ This technique enables a shorter insertion time but requires a sufficient number of years of training to master fully. Although trainees exhibited improvement, they still fell short of the proficiency levels of the attendings, implying that additional training time may be required for them to attain full proficiency in insertion. Improvement in PE, particularly pronounced during the third year, may be attributed to trainees gaining proficiency by performing several procedures with varying degrees of difficulty. The majority of post‐treatment surveillance and high‐risk screening colonoscopies at our cancer center contributed to the high ADR (47%) observed in first‐year trainees. Despite the overall improvement over time, the lack of significant differences may be because of the distinctive characteristics of these cases, and the fact that the proportion of surveillance colonoscopies with a high risk of detecting iatrogenic lesions was greater with the first‐ and second‐year trainees than with the third‐year trainees.^16^ Moreover, the improvement in MAP over time and the sub‐analysis of surveillance colonoscopy showed significant improvements, suggesting that our advanced fellowship is effective in enhancing competency to detect lesions. In the sub‐analysis based on the number of colonoscopies performed prior to beginning the advanced fellowship, the improvement in PE was observed only among trainees with <500 colonoscopy experiences. This result is similar to that of a previous report that calculated the number of cases required to achieve total colonoscopy competence,^17^ suggesting that approximately 500 cases are required to attain proficiency in lesion detection and polypectomy procedures.^4^, ^18^ This study's findings suggest that long‐term training is necessary for trainees to become independent endoscopists in colonoscopy.

This study has several limitations. First, it was a retrospective observational study and the uneven distribution of colonoscopy indications over the years may have influenced the evaluation of these indicators. Additionally, one‐on‐one instruction was enforced only during the first and second years, we could not entirely exclude the possibility that the absence of one‐on‐one instruction during the third year may have contributed to an apparent improvement in PE. Second, differences in scope, light source, and extraction modality were not considered. The factors that can significantly impact insertion time and treatment efficiency,^19^ and light source and image modality, as typified by image‐enhanced endoscopy, affect lesion detection rates, such as ADR^20^; their impact on outcomes could not be evaluated. Third, assessing whether the observed improvement in trainees' skills in this study was attributable to the program itself or to individual growth over time remains a complex task.

In conclusion, this study suggests that trainees after completing a general fellowship have shown improvements in their ability to perform higher‐quality colonoscopies under our advanced endoscopy fellowship.

## CONFLICT OF INTEREST STATEMENT

Author Hiroaki Ikematsu is an associate editor of DEN Open. The other authors declare no conflict of interest.

## ETHICS STATEMENT

This study was approved by the institutional research board of the National Cancer Center Hospital East

## PATIENT INFORMED CONSENT

N/A.

## CLINICAL TRIAL REGISTRATION

Research Project No. 2017–434

## Supporting information

TABLE S1 Breakdown of colonoscopies performed during the study period.
